# Free Immunoglobulin Light Chains in Patients with Tick-Borne Encephalitis: Before and after Treatment

**DOI:** 10.3390/jcm10132922

**Published:** 2021-06-29

**Authors:** Monika Gudowska-Sawczuk, Piotr Czupryna, Anna Moniuszko-Malinowska, Sławomir Pancewicz, Barbara Mroczko

**Affiliations:** 1 Department of Biochemical Diagnostics, Medical University of Bialystok, ul. Waszyngtona 15A, 15-269 Bialystok, Poland; mroczko@umb.edu.pl; 2 Department of Infectious Diseases and Neuroinfections, Medical University of Bialystok, ul. Żurawia 14, 15-540 Bialystok, Poland; avalon-5@wp.pl (P.C.); annamoniuszko@op.pl (A.M.-M.); spancewicz@interia.pl (S.P.); 3 Department of Neurodegeneration Diagnostics, Medical University of Bialystok, ul. Waszyngtona 15A, 15-269 Bialystok, Poland

**Keywords:** free light chains, lambda, kappa, tick-borne encephalitis, biomarker

## Abstract

Background: Tick-borne encephalitis (TBE) is inflammation of the central nervous system (CNS) caused by a viral infection which may be associated with increased synthesis of immunoglobulins. It can lead to inter alia, breakdown of the blood-brain barrier (BBB), or even death and, unfortunately, treatment is only symptomatic. Therefore, the aim of the present study was assessment of the concentrations of free light chains (FLC) kappa (κ) and lambda (λ in the cerebrospinal fluid (CSF) and serum of patients with TBE. Methods: A total of 58 cerebrospinal fluid and serum sample pairs were analyzed. Samples were collected from patients with TBE before and after treatment. FLC were measured using the turbidimetric method. The values of κIgG-index, λIgG-index, κFLC-index and λFLC-index were calculated using relevant formulas. Results: Pre-treatment serum λFLC concentrations were higher in comparison to post-treatment levels. Moreover, it was observed that CSF λFLC, TBEV IgM, TBEV IgG, and serum TBEV IgG, as well as the values of λFLC-index, κFLC-index, and λIgG-index were elevated after treatment. In the total study group, the concentrations of CSF κFLC and λFLC, and values of four indexes: κFLC-index, λFLC-index, κIgG-index, and λIgG-index correlated with each other and with CSF TBEV IgM and IgG antibodies. The CSF level of TBEV IgG was also associated with serum IgG TBEV and CSF IgM TBEV antibodies. Additionally, serum κFLC correlated with serum and CSF λFLC. Conclusion: This is the first study that demonstrates statistically significant differences in serum and CSF λFLC, as well as in the calculated values of three algorithms: λIgG-index, κFLC-index, and λIgG-index prior to and following treatment of TBE. Our findings may indicate that these differences reflect the intrathecal synthesis of immunoglobulins and increased permeability of BBB in patients with TBE. Moreover, it could provide the basis for developing new therapeutic strategies.

## 1. Introduction

The simplest definition of encephalitis is “brain inflammation”. The most common causes of encephalitis are viral infections [1]. Tick-borne encephalitis (TBE) is a growing health problem, particularly in forested regions of Europe and Asia [2]. TBE is an infection of the central nervous system which can cause mild to severe illness, such as breakdown of the blood-brain barrier (BBB) or permanent neurological complications, and at times can lead to death. The tick-borne encephalitis virus (TBEV) is the sole member of the genus Flavivirus in the family Flaviviridae. On average, symptoms of tick-borne encephalitis appear around 7–14 days after a bite from an Ixodes spp. tick and virus replication [3,4]. The severity can be variable and the disease most often manifests as encephalitis, meningitis, or meningoencephalitis [5].

The diagnosis of TBE is based on the identification and determination of TBEV-specific antibodies. Serological examination of the serum and cerebrospinal fluid (CSF), obtained by lumbar puncture, is performed. Inflammatory changes in CSF last for a few weeks, and less frequently, several months. The concentration of TBEV-specific IgM and IgG antibodies in the serum and CSF is measured, and the results are compared with the diagnostic threshold value. The production and presence of specific IgM antibodies, usually lasting up to six months, is characteristic of the acute phase of the disease. Specific IgG antibodies persist for years and prove the obtained resistance to TBEV [6,7]. Furthermore, it is well known that human immunoglobulins consist of four polypeptide chains: two heavy and two light chains kappa (κ) or lambda (λ). Light chains are always produced in excess in comparison to heavy ones during the synthesis of immunoglobulins. Light chains that are not combined with heavy chains are called free light chains (FLC). Therefore, an elevated concentration of free light chains may indicate increased synthesis of immunoglobulins [8,9].

Currently, there is no causal treatment for TBE. Even severe forms of the disease are treated only symptomatically with anti-inflammatory drugs that reduce brain swelling, antipyretics, and painkillers [10]. There are some studies confirming the response to the treatment using free light chains concentrations, e.g., in multiple myeloma or amyloidosis [11,12]. However, to our knowledge, this is the first study that evaluated the clinical significance of FLC in tick-borne encephalitis. In the present paper, we investigated changes of ongoing dynamics of kappa and lambda free light chain concentrations in serum and CSF. Moreover, four indexes (κIgG-index, λIgG-index, κFLC-index, and λFLC-index) were calculated on the basis of serum and CSF FLC concentrations prior to, and following, treatment.

## 2. Material and Methods

### 2.1. Subjects

Patients admitted to the Department of Infectious Diseases and Neuroinfections at the Medical University of Bialystok constituted the study group. The participants underwent a lumbar puncture for diagnostic purposes. 

A total of 58 cerebrospinal fluid and serum sample pairs were collected from 29 patients with TBE (18 males and 11 females, age range 35–74 years). A total of 29 paired samples were obtained upon admission (beginning of the neurological phase of TBE) and upon discharge—following 15.9 ± 6.2 days of hospitalization (patients who recovered and no longer needed hospital treatment). On the basis of inflammatory parameters in the CSF with no focal neurological symptoms, thirteen patients were diagnosed with meningitis. On the basis of inflammatory parameters in the CSF, altered consciousness and, presence of focal neurological symptoms, sixteen patients were diagnosed with meningoencephalitis. Patient characteristics are presented in Table 1.

The diagnosis was based on clinical data and presence of inflammatory parameters in the CSF. To confirm TBE, the presence of TBEV specific antibodies in serum and CSF was evaluated. 

Patients were treated with Mannitol at a dose of 0.25 g/kg body weight as a 15% solution administered 2–4 times per day. In five patients with meningoencephalitis, Dexamethasone, at a dose of 4–24 mg per day for 4–6 days, was used. The main analgesic used was Acetaminophen.

Written informed consent was obtained from all study participants. The study was approved by the Bioethics Committee at the Medical University of Bialystok.

### 2.2. CSF and Blood Sampling

CSF samples were collected into polypropylene tubes and venous blood samples were obtained by venipuncture. Venous blood samples were centrifuged to separate the serum. Both CSF and serum samples were aliquoted and frozen at −80 °C until assayed. The samples were collected prior to (sample 1) and following treatment (sample 2). Changes in the tested biomarkers before and after treatment were examined in all patients.

κFLC, λFLC, albumin, and IgG in the CSF and serum were measured on the Optilite analyzer (The Binding Site) according to the turbidimetric method. When FLC concentrations were below the limit of detection, we used the corresponding detection limit: CSF κFLC—0.30 mg/L and CSF λFLC—0.65 mg/L.

TBEV specific antibodies titer was measured using the Enzygnost Anti-TBE/FSME Virus (IgG, IgM) Siemens test (OD korr./U).

### 2.3. Calculations

Results were also expressed as κIgG-index, λIgG-index, κFLC-index and λFLC-index. Indexes were calculated according to the following formulas:

$$\kappa \mathrm{IgG}-\mathrm{index}=\frac{\mathrm{CSF}\;\kappa \mathrm{FLC}\;\left(\frac{\mathrm{mg}}{L}\right)/\mathrm{serum}\;\kappa \mathrm{FLC}\left(\frac{\mathrm{mg}}{L}\right)}{\mathrm{CSF}\;\mathrm{IgG}\;\left(\frac{\mathrm{mg}}{L}\right)/\mathrm{serum}\;\mathrm{IgG}\left(\frac{g}{L}\right)}\times 100$$

$$\lambda \mathrm{IgG}-\mathrm{index}=\frac{\mathrm{CSF}\;\lambda \mathrm{FLC}\;\left(\frac{\mathrm{mg}}{L}\right)/\mathrm{serum}\;\lambda \mathrm{FLC}\left(\frac{\mathrm{mg}}{L}\right)}{\mathrm{CSF}\;\mathrm{IgG}\;\left(\frac{\mathrm{mg}}{L}\right)/\mathrm{serum}\;\mathrm{IgG}\left(\frac{g}{L}\right)}\times 100$$

$$\kappa \mathrm{FLC}-\mathrm{index}=\frac{\mathrm{CSF}\;\kappa \mathrm{FLC}\left(\frac{\mathrm{mg}}{L}\right)/\mathrm{serum}\;\kappa \mathrm{FLC}\left(\frac{\mathrm{mg}}{L}\right)}{\mathrm{CSF}\;\mathrm{albumin}\left(\frac{\mathrm{mg}}{L}\right)/\mathrm{serum}\;\mathrm{albumin}\left(\frac{\mathrm{mg}}{L}\right)}$$

$$\lambda \mathrm{FLC}-\mathrm{index}=\frac{\mathrm{CSF}\;\lambda \mathrm{FLC}\left(\frac{\mathrm{mg}}{L}\right)/\mathrm{serum}\;\lambda \mathrm{FLC}\left(\frac{\mathrm{mg}}{L}\right)}{\mathrm{CSF}\;\mathrm{albumin}\left(\frac{\mathrm{mg}}{L}\right)/\mathrm{serum}\;\mathrm{albumin}\left(\frac{\mathrm{mg}}{L}\right)}$$

The κIgG-index and λIgG-index has been developed by Gudowska-Sawczuk et al. whereas the κFLC-index and λFLC-index has been developed by Presslauer S et al. [13,14].

### 2.4. Statistical Analysis

Statistical analysis was performed using Statistica 13.3 (TIBCO Software, Palo Alto, CA, USA). Differences between the tested groups were evaluated by the Mann-Whitney U test. The Spearman rank correlation coefficient was used to measure the degree of association between variables. We considered *p* < 0.05 as statistically significant.

## 3. Results

The results of routine laboratory tests in patients with TBE are presented in Table 2. Statistically significant differences prior to (sample 1) and following (sample 2) treatment in the Mann Whitney U test were observed for the concentration of serum IgG, CSF cytosis, and the percentage of multinuclear cells and lymphocytes present in the CSF (*p* = 0.020; *p* < 0.001; *p* < 0.001; *p* < 0.001, respectively).

The results of free light chains concentrations and specific TBEV antibodies, in patients with TBE, are presented in Table 3. The concentrations of serum λFLC were markedly elevated before treatment (*p* = 0.007) while the concentrations of CSF λFLC were markedly elevated after treatment (*p* = 0.047). The concentrations of serum and CSF κFLC did not differ between sample 1 and sample 2 (*p* = 0.095; *p* = 0.055. respectively). The values of serum TBEV IgG. CSF TBEV IgM and CSF TBEV IgG were higher after treatment (*p* < 0.001; *p* = 0.019; *p* = 0.044, respectively). The values of serum TBEV IgM were similar in sample 1 and sample 2 (*p* = 0.449).

**Figure 1:**
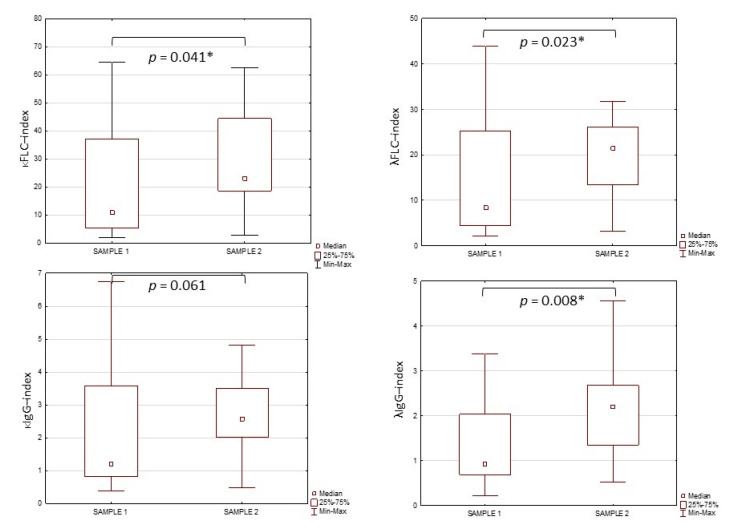
The values of κFLC-index, λFLC-index, κIgG-index and λIgG-index in tested groups. *—significant differences between tested groups (*p* < 0.05).

Differences in the κFLC-index, λFLC-index, κIgG-index and λIgG-index values in the tested groups are presented in Figure 1. The values of κFLC-index, λFLC-index and λIgG-index were significantly higher in sample 2 (31.75 ± 21.79; 27.15 ± 20.80; 2.42 ± 1.42. respectively) compared to sample 1 (29.68 ± 54.28; 22.87 ± 37.34; 2.37 ± 1.73. respectively). There were no differences in the κIgG-index values between the tested groups (2.09 ± 4.65 vs. 2.42 ± 1.47).

There were no significant differences in S κFLC, CSF κFLC, S λFLC, CSF λFLC, κFLC-index, λFLC-index, κIgG-index, λIgG-index between patients with meningitis and those with meningoencephalitis prior to treatment (*p* = 0.843; *p* = 0.080; *p* = 0.293; *p* = 0.384; *p* = 0.084; *p* = 0.849; *p* = 0.341; *p* = 0.265, respectively). There were no significant differences in S κFLC, CSF κFLC, S λFLC, CSF λFLC, κFLC-index, λFLC-index, κIgG-index, λIgG-index between patients with meningitis and those with meningoencephalitis following treatment (*p* = 0.890; *p* = 0.368; *p* = 0.580; *p* = 0.180; *p* = 0.221; *p* = 0.053; *p* = 0.221; *p* = 0.935, respectively).

Correlations between CSF κFLC, CSF λFLC, κFLC-index, λFLC-index, κIgG-index, λIgG-index and specific TBEV antibodies are presented in Table 4. The Spearman’s rank correlation test demonstrated that, in the total study group, CSF κFLC, CSF λFLC, κFLC-index, λFLC-index, κIgG-index, and λIgG-index correlated with each other. In addition, the parameters mentioned above correlated with CSF TBEV IgM and IgG antibodies. CSF TBEV IgG correlated with serum IgG and CSF IgM TBEV antibodies. We also found a positive correlation between serum κFLC, and serum and CSF λFLC.

## 4. Discussion

Following contact with an infected tick, the virus initially multiplies in the skin and lymph nodes, and it enters blood through the lymphatic system. In the primary phase of viremia, cells of various organs are infected but, at the same time, the immunological system is activated. Due to immunological mechanisms, viruses are eliminated. Cytotoxic T lymphocytes, which recognize and kill virus-infected cells, play a major role in this process [15,16]. However, B-cells are as important as T lymphocytes due to their ability to produce specific immunoglobulins that may directly neutralize the virus [17]. It is well established that, during the synthesis of immunoglobulins, light chains are synthesized in excess in relation to heavy chains. The excess light chains that are not part of the whole immunoglobulin are called free light chains [8,9].

There is currently no specific treatment for TBE, and medication can only help to control symptoms. Additionally, it has been revealed that an elevated lymphocyte count may persist for a number of weeks following treatment [18]. Therefore, the present study examined the relationship between serum and CSF concentrations of free light chains and the treatment of symptomatic TBE.

The first sample was obtained from each study participant on admission to hospital, usually a short time after the beginning of the second (neurological) phase of TBE, while the second sample was collected in the recovery phase (disappearance of acute disease symptoms), prior to the patient’s discharge from hospital.

In the present study, we observed that serum λFLC concentrations were significantly elevated prior to treatment in comparison to post-treatment values. Furthermore, in sample 1, the levels of serum total IgG were also increased. Therefore, elevated λFLC levels are presumably caused by enhanced production of immunoglobulins during inflammation in patients with TBE. On the other hand, the concentrations of CSF λFLC were elevated following treatment and were almost twice as high as serum λFLC levels.

It is well established that serum is the main source of proteins present in the CSF and the protein level is regulated by, inter alia, the permeability of the blood-brain-barrier and the CSF flow rate. Enhanced levels of immunoglobulins in the CSF may indicate increased permeability of BBB or intrathecal synthesis in the CNS. Additionally, FLC exists in two major molecular forms: monomers or dimers. λFLC are often dimers which, in normal conditions, should not pass the blood-brain barrier [19]. Therefore, presence of λFLC in the CSF may indicate intrathecal immunoglobulin synthesis or blood-brain barrier (BBB) dysfunction. Knowing that neuroinflammation can lead to BBB damage [20], we speculated that, in patients with TBE, the presence of λFLC in the CSF may be caused by the fact that lambda free light chain cells pass the leaky blood-brain barrier. Furthermore, we observed that, following treatment, the percentage of CSF lymphocytes was higher and λFLC concentrations correlated with CSF TBEV IgM and IgG. Knowing that free light chains are produced regardless of the type of heavy chains, it can suggest that increased levels of lambda light chains are associated with enhanced synthesis of specific antibodies, both IgM and IgG, by lymphocytes. Therefore, elevated post-treatment λFLC levels may be an indicator of increased BBB permeability. Thus, we carefully suggest that lambda free light chains can predict severe sequelae of tick-borne encephalitis.

Consequently, in this study, we examined values of four indexes calculated on the basis of λFLC, κFLC, albumin, and IgG concentrations: κFLC-index, λFLC-index, and those developed in our previous study: λIgG-index and κIgG-index [13]. We showed that the values of κFLC-index, λFLC-index, and λIgG-index were significantly higher following treatment. Elevation of κFLC-index and λFLC-index is in accordance with other studies that reported differences in the values of κFLC-index and λFLC-index between multiple sclerosis (MS), which is also a neuroinflammatory condition, and other non-inflammatory neurological disorders [13,14,21,22]. Moreover, in this study, we observed that λIgG-index values were markedly elevated in sample 2. By contrast, our previous study revealed that λIgG-index was similar in MS and other non-inflammatory neurological disorders, but patients with MS had elevated κIgG-index [13]. Therefore, we speculate that, probably, only λIgG-index will be useful in differentiating TBE from other conditions involving the central nervous system.

The limitation of the present study was a relatively small sample size and, therefore, future studies should aim to confirm the clinical and prognostic role of free light chains in tick-borne encephalitis in a larger population. 

The results of our current study confirm that there are differences in serum and CSF concentrations of λ free light chains differ between the initial stage of the disease and following treatment. A decrease in serum λFLC, and an increase in CSF λFLC, after treatment may reflect the intrathecal synthesis of immunoglobulins or the blood-brain barrier disruption.

## Figures and Tables

**Table 1:** Characteristics of TBE patients according to the course of disease.

	Meningitis	Meningoencephalitis
Demographics		
*n*	13	16
sex	4 female, 9 male	7 female, 9 male
age (years)	55.5 ± 10.6	54.5 ± 8.9
hospitalization (days)	14.8 ± 3.7	16.9 ± 7.8
	Complaints	
fever	13 (100%)	14 (87.5%)
headache	13 (100%)	16 (100%)
vomitting	1 (7.7%)	1 (6.3%)
nausea	6 (46.2%)	5 (31.3%)
vertigo	2 (15.4%)	6 (37.5%)
Neurological Symptoms		
tremor	0	5 (31.3%)
balance disorder	0	5 (31.3%)
paresis	0	2 (12.5%)
consciousness disturbances	0	16 (100%)
Comorbidities		
hypertension	1 (7.7%)	0
diabetes	0	1 (6.3%)

**Table 2:** Results of routine laboratory tests in patients with tick-borne encephalitis.

	Alb. S (g/L)	Alb. CSF (mg/L)	Q_Alb_	IgG S (g/L)	IgG CSF (mg/L)	Q_IgG_	CRP (mg/L)	CSF Cytosis (cells/Ml)	CSF Mono. Cells (%)	CSF Multi. Cells (%)	CSF Lymph. (%)
Sample 1 before Treatment	54.30 (35.30–72.10)	345.60 (202.40–1893.80)	6.44 (3.77–38.03)	14.89 * (10.63–21.87)	80.49 (26.32–365.45)	5.72 (2.06–24.95)	2.79 (1.00–29.05)	96 * (4–591)	65 (9.1–98.3)	33.6 * (1–90.1)	43 * (9.1–98.3)
Sample 2 after Treatment	50.35 (36.40–71.60)	305.25 (195.80–1979.10)	6.44 (2.99–29.33)	13.15 (6.33–21.16)	94.99 (32.66–430.23)	7.62 (3.22–27.86)	1.60 (0.30–27.75)	23 (3–93)	89.1 (17–100)	2 (0–20)	97 (96–98)
*p-*Value	0.079	0.596	0.970	0.020	0.890	0.381	0.171	<0.001	0.081	<0.001	<0.001

Data are median values (min.-max.); *—significant differences in comparison to Sample 2 (after treatment) (*p* < 0.05). Alb. Albumin; Mono. Mononuclear; Multi. Multinuclear; Lymph. Lymphocytes; S. serum; CSF. cerebrospinal fluid.

**Table 3:** Results of specific laboratory tests and calculated indexes in patients with tick-borne encephalitis.

Variable Tested								
Median (Min–Max Values)								
	κFLC S (mg/L)	λFLC S (mg/L)	κFLC CSF (mg/L)	λFLC CSF (mg/L)	Serum TBEV IgM (OD korr./U)	Serum TBEV IgG (OD korr./U)	CSF TBEV IgM (OD korr./U)	CSF TBEV IgG (OD korr./U)
Sample 1 before Treatment	22.06 (11.43–39.23)	24.26 * (13.54–55.75)	2.33 (0.31–13.04)	1.39 * (0.70–23.73)	112.70 (1.70–154.2)	370.60 * (8.60–1461.70)	53.80 * (0.60–160.90)	335.20 * (2.50–3079.40)
Sample 2 after Treatment	19.96 (10.63–30.79)	18.23 (12.42–29.00)	3.66 (0.62–13.04)	2.69 (0.70–38.22)	93.30 (22.60–197.00)	1541.45 (679.80–3046.60)	148.30 (21.30–195.30)	1204.00 (601.40–3086.30)

S, serum; CSF, cerebrospinal fluid. *—significant differences between tested groups (*p* < 0.05).

**Table 4:** The Spearman’s correlations between tested variables in the total study group.

Total Study Group	S-κ	S-λ	CSF-κ	CSF-λ	κFLC-index	λFLC-index	κIgG-index	λIgG-index	S TBEV IgM	CSF TBEV IgM	S TBEV IgG	CSF TBEV IgG
S-κ												
r		**0.738**	0.009	**0.738**	−0.224	−0.061	0.269	−0.109	0.259	−0.283	0.171	−0.187
p		<0.001 *	0.951	<0.001 *	0.1	0.662	0.047	0.434	0.192	0.213	0.394	0.417
S-λ												
r	**0.738**		−0.085	−0.158	−0.081	−0.207	−0.201	**−0.342**	0.212	−0.127	−0.029	−0.19
p	<0.001 *		0.536	0.25	0.555	0.133	0.14	0.011 *	0.289	0.583	0.887	0.41
CSF-κ												
r	0.009	−0.085		**0.844**	**0.813**	**0.635**	**0.758**	**0.471**	0.058	**0.634**	0.055	**0.496**
p	0.951	0.536		<0.001 *	<0.001 *	<0.001 *	<0.001 *	<0.001 *	0.788	0.003 *	0.799	0.022 *
CSF-λ												
r	**0.738**	−0.158	**0.844**		**0.73**	**0.858**	**0.697**	**0.741**	0.224	**0.642**	0.21	**0.592**
p	<0.001 *	0.25	<0.001 *		<0.001 *	<0.001 *	<0.001 *	<0.001 *	0.304	0.002 *	0.336	0.001 *
κFLC-index												
r	−0.224	−0.081	**0.813**	**0.73**		**0.775**	**0.897**	**0.574**	−0.04	**0.651**	−0.059	**0.532**
p	0.1	0.555	<0.001 *	<0.001 *		<0.001 *	<0.001 *	<0.001 *	0.853	<0.001 *	0.784	0.016 *
λIgG-index												
r	−0.061	−0.207	**0.635**	**0.858**	**0.775**		**0.733**	**0.879**	0.115	**0.63**	0.128	**0.672**
p	0.662	0.133	<0.001 *	<0.001 *	<0.001 *		<0.001 *	<0.001 *	0.601	0.003 *	0.559	0.001 *
κIgG-index												
r	0.269	−0.201	**0.758**	**0.697**	**0.897**	**0.733**		**0.71**	−0.047	**0.554**	−0.164	**0.459**
p	0.047	0.14	<0.001 *	<0.001 *	<0.001 *	<0.001 *		<0.001 *	0.826	0.011 *	0.443	0.042 *
λIgG-index												
r	−0.109	**−0.342**	**0.471**	**0.741**	**0.574**	**0.879**	**0.71**		0.077	**0.551**	0.165	**0.668**
p	0.434	0.011 *	<0.001 *	<0.001 *	<0.001 *	<0.001 *	<0.001 *		0.727	0.012 *	0.452	0.001 *
S TBEV IgM												
r	0.259	0.212	0.058	0.224	−0.04	0.115	−0.047	0.077		0.492	0.115	0.225
p	0.192	0.289	0.788	0.304	0.853	0.601	0.826	0.727		0.087	0.567	0.459
CSF TBEV IgM												
r	−0.283	−0.127	**0.634**	**0.642**	**0.651**	**0.63**	**0.554**	**0.551**	0.492		0.55	**0.727**
p	0.213	0.583	0.003 *	0.002 *	<0.001 *	0.003 *	0.011 *	0.012 *	0.087		0.051	<0.001 *
STBEV IgG												
r	0.171	−0.029	0.055	0.21	−0.059	0.128	−0.164	0.165	0.115	0.55		**0.786**
p	0.394	0.887	0.799	0.336	0.784	0.559	0.443	0.452	0.567	0.051		0.001 *
CSF TBEV IgG												
r	−0.187	−0.19	**0.496**	**0.592**	**0.532**	**0.672**	**0.459**	**0.668**	0.225	**0.727**	**0.786**	
p	0.417	0.41	0.022 *	0.001 *	0.016 *	0.001 *	0.042 *	0.001 *	0.459	<0.001 *	0.001 *	

S, serum; CSF, cerebrospinal fluid. * or bold—significant coorelation between tested parameters (*p* < 0.05).

## Data Availability

The data that support the findings will be available on request under the corresponding author’s e-mail: monika.gudowska-sawczuk@umb.edu.pl.
